# Intradural Disk Herniation Mimicking a Spinal Tumor: Radiologic Imaging, Pathogenesis, and Operative Management

**DOI:** 10.1155/2018/9810762

**Published:** 2018-04-29

**Authors:** Daisuke Tateiwa, Ryoji Yamasaki, Rinsei Tei, Yasushi Shin, Kenta Ariga, Kenji Hayashida, Eiji Wada

**Affiliations:** ^1^Department of Orthopaedic Surgery, Osaka Police Hospital, 10-31 Kitayama-cho, Tennoji-ku, Osaka 543-0035, Japan; ^2^Spine and Spinal Cord Center, Osaka Police Hospital, 10-31 Kitayama-cho, Tennoji-ku, Osaka 543-0035, Japan

## Abstract

Intradural disk herniation (IDH) is a rare condition, occurring more often at the L4-5 level. We examined a case of an IDH at the L1-2 level mimicking an intradural spinal tumor. A 71-year-old woman with a long history of backache and pain radiating down the left leg was admitted to our hospital with the worsening of these symptoms. Magnetic resonance imaging and computed tomographic myelography demonstrated an intradural mass at the L1-2 level. Given the radiologic findings and the location of the mass, the preoperative differential diagnosis centered on intradural spinal tumors. Dural incision was performed using a surgical microscope to resect the mass. Contrary to our expectation, the diagnosis made during the surgery was IDH. Despite advances in imaging techniques, IDH could not be definitively diagnosed preoperatively. The pathogenesis of IDH remains unclear. In our patient, the ventral dural defect was smooth and round, and the dural tissue around the defect was thickened. These intraoperative findings suggested that the patient's IDH resulted not from an acute new event but from a chronic process. We recommend dural incision using a surgical microscope for treating IDH because it provides a clear visual field.

## 1. Introduction

Intradural disk herniation (IDH) is a rare condition, comprising 0.26%–0.30% of all disk herniations [1, 2]. It occurs at the lumbar level in 92% of the patients [1, 2], with the L4-5 disk space being the most commonly affected site (55%), followed by L3-4 (16%), L5-S1 (10%), and L2-3 and L1-2 [3]. Thus, IDH at the L1-2 level is particularly rare. Despite advances in neuroimaging techniques, such as computed tomography (CT) and magnetic resonance imaging (MRI), a preoperative diagnosis of IDH is difficult because it is easily confused with other spinal abnormalities, such as schwannoma, ependymoma, neurofibroma, meningioma, epidermoid tumor, arachnoid cyst, metastasis, and subdural abscess. Therefore, in most patients, the final diagnosis of IDH is only made intraoperatively [4]. We present the case of a patient with IDH at the L1-2 level mimicking intradural spinal tumor.

## 2. Case Presentation

### 2.1. History

A 71-year-old woman suffered from backache and pain radiating down her left leg for 7 years. She had been treated at several clinics, but her symptoms persisted, with repeated cycles of exacerbation and remission. She was admitted to our hospital with a rapid worsening of the backache and left leg pain and a new onset of pain in the right leg and bilateral leg weakness for 3 months before admission. She had no history of trauma or previous lower back surgery. Neurologic examination revealed grade 1/5 motor power in the hip flexors and grade 3/5 power in the knee and ankle extensors and extensor and flexor hallucis longus bilaterally. Straight leg raising was positive at 30° bilaterally. There was a sensory deficit involving both legs and the genital area. Her bladder and bowel control was reportedly normal. Because she was in severe pain and could not walk well as a result of the motor deficit, she agreed to undergo surgery.

### 2.2. Preoperative Imaging

**Figure 1:**
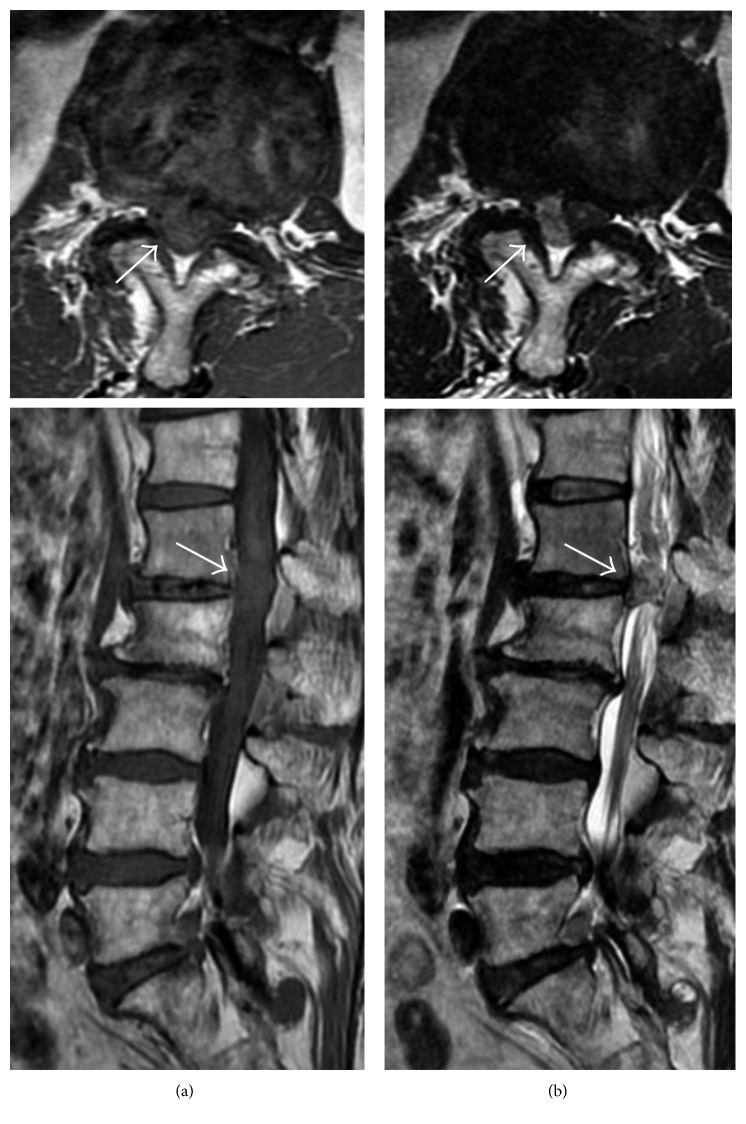
T1- and T2-weighted images from noncontrast magnetic resonance imaging: a mass is seen at the L1-2 level (arrow) with a low intensity on T1-weighted imaging (a) and a slightly high intensity on the T2-weighted image (b). The axial views demonstrate the mass occupying the right side of the spinal canal (arrow).

**Figure 2:**
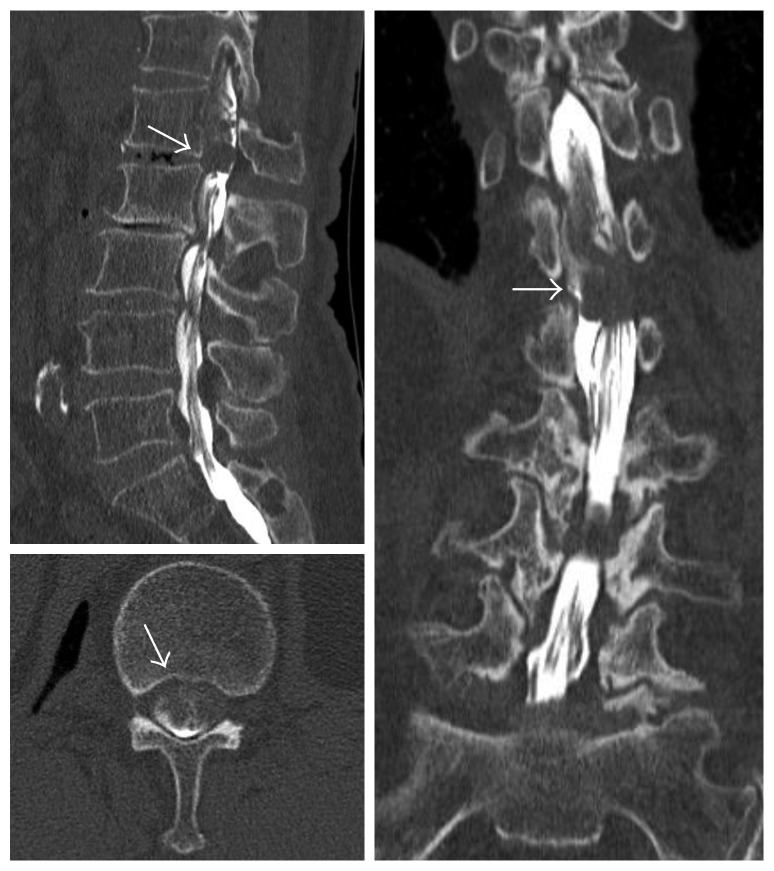
Computed tomographic myelography demonstrates the mass located in the right side of the intradural space, significantly compressing the cauda equina (arrow).

**Figure 3:**
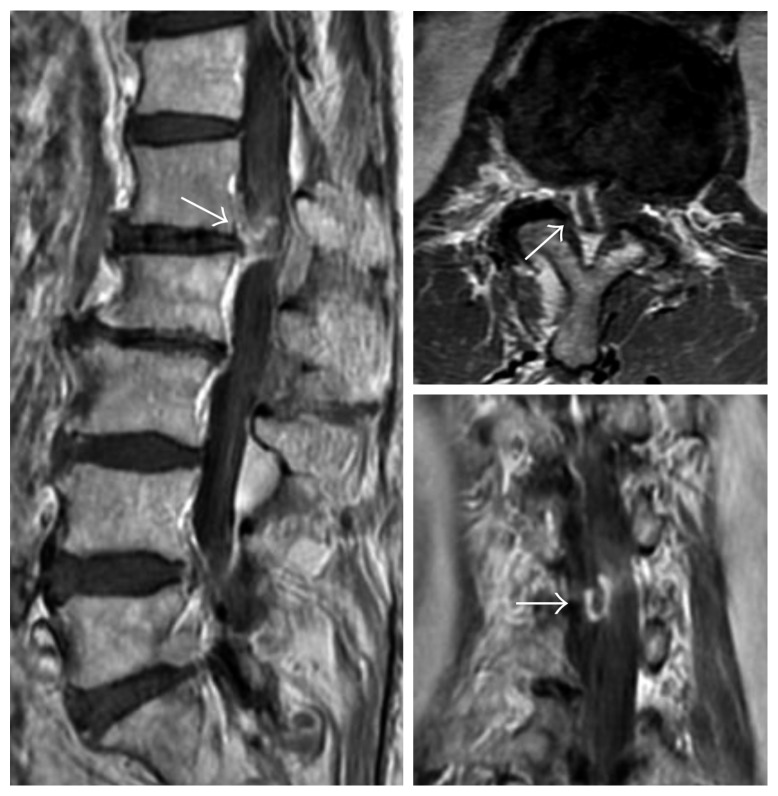
Contrast-enhanced magnetic resonance imaging: T1-weighted images show a peripheral rim enhancement of the intradural lesion (arrow).

MRI revealed an inhomogeneous mass at the L1-2 level with a low intensity on T1-weighted imaging and a slightly high intensity on T2-weighted imaging. An axial slice showed that the mass occupied the right side of the spinal canal (Figure 1), but it was unclear whether it was located in the extradural or intradural space. CT myelography clearly revealed that the mass was intradural and was compressing the cauda equina nerve rootlets (Figure 2). On contrast-enhanced MRI, T1-weighted images showed a rim enhancement of the mass (Figure 3). No clear discontinuity of the posterior longitudinal ligament (PLL) was seen on both noncontrast- and contrast-enhanced MRI.

The radiologic findings, such as the rim enhancement on MRI, suggested a differential diagnosis of IDH, schwannoma, ependymoma, or metastasis. However, because a discontinuity of the PLL was not observed and the location of the IDH at the L1-2 level was unusual, we assumed that the diagnosis of LDH was unlikely.

### 2.3. Operation and Postoperative Course

**Figure 4:**
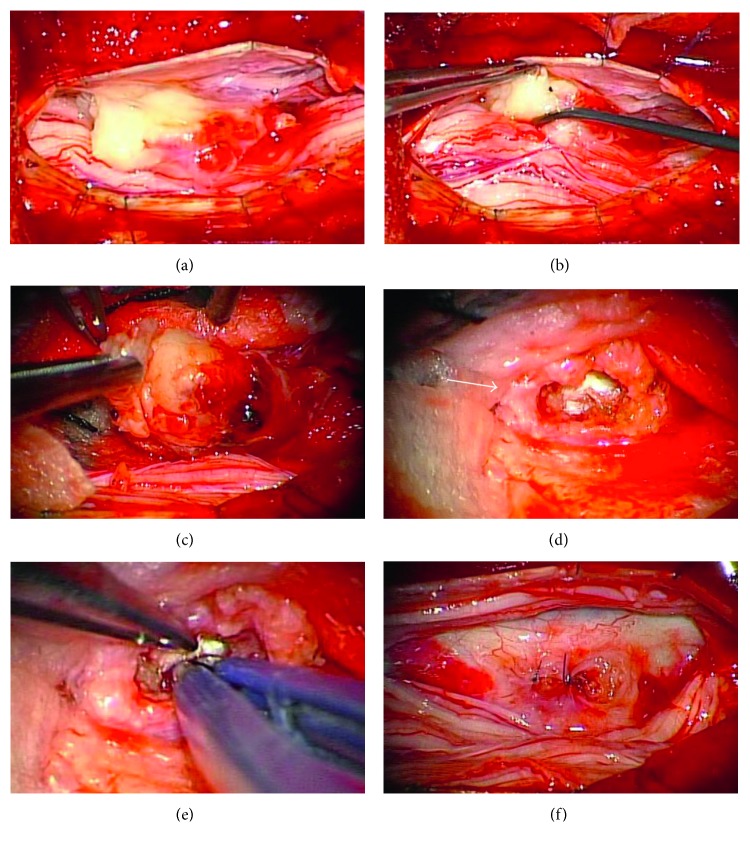
(a) On dorsal dural incision, a mass is observed between the nerve rootlets. (b) A dissector and sucker were used to carefully peel the adherent nerve rootlets away from the disk fragment. (c) The disk fragment was resected by the piecemeal technique. (d) A round defect of the ventral dura and a tear in the disk annulus are shown. The dural tissue around the defect is thickened (arrow). (e) The tear in the annulus was confirmed by inserting the tip of bipolar forceps into the tear. (f) After trimming the thickened margin, the dural defect was repaired using nylon sutures.

The surgery was performed using a surgical microscope. Subtotal laminectomy was performed at the L1 and L2 levels. No abnormality was seen in the epidural space, which was expected based on our preoperative differential diagnosis. A hard mass lesion was palpable within the dural sac. After dorsal dural incision was performed, we found a yellowish, rubbery, solid mass which was adherent to the cauda equina nerve roots within the subarachnoid space (Figure 4(a)). An examination of an intraoperative frozen section showed the mass to be a disk fragment. The nerve roots adhering to the fragment were carefully dissected using a dissector and sucker (Figure 4(b)), and the fragment was resected by the piecemeal technique (Figure 4(c)). We found a smooth and round defect of the ventral dura and a tear in the disk annulus (Figures 4(d) and 4(e)). The dural tissue around the defect was thickened and adherent to the PLL. After trimming the thickened margin around the defect, we closed it using nylon sutures (Figure 4(f)). The dorsal dural closure was performed using nonpenetrating titanium clips, and soft tissues were ordinarily closed.

One month postoperatively, the patient reported a significant relief of the pain and an almost complete recovery of the motor deficit. The numbness regressed, but slightly persisted.

## 3. Discussion

Our case illustrates the difficulty of preoperatively diagnosing IDH, its chronic pathogenesis, and a successful operative management of the condition.

Despite advances in imaging techniques, there are challenges associated with the preoperative diagnosis of IDH. Several authors have reported the usefulness of MRI in neuroimaging studies for the diagnosis of IDH [4, 5]. After conducting imaging diagnosis, our case did not reveal a discontinuity of the PLL and a “hawk-beak sign” on MRI, which have been reported to be helpful in diagnosing IDH [4, 6]. One of the most common radiological signs seen in IDH is the rim enhancement, which was observed in our patient on the contrast-enhanced MRI [7]. This sign is thought to be the result of the infiltration of the vascular granulation tissue into the lesion [8]. However, rim enhancement is not specific for IDH because it has also been reported in schwannoma [9], metastasis [10], ependymoma [11], subdural abscess [12], and extradural disk herniation (EDH) [4]. Therefore, given the radiological findings of the mass and its rare location, we believed that IDH was not the likely explanation for our patient's lesion. It was only during the surgery that the correct diagnosis was made.

The pathogenesis of IDH remains unclear, but there is general agreement that anatomic adhesions between the ventral dura and the PLL are significant predisposing factors [13–15]. These adhesions may be congenital [15] or may occur later in life because of degenerative disk disease [16], previous spine surgery, or trauma [16–18]. However, the process of herniated disk penetrating the dura adherent to the PLL is unknown. In our patient, the ventral dural defect was smooth and round, and the dural tissue around the defect was thickened. Considering these intraoperative findings and the patient's long-term clinical history of symptoms, we suspected that her IDH resulted not from an acute new event but from a chronic process. When a herniated disk caused repetitive pressure on the dura adherent to the PLL, the disk could not escape into the space between the PLL and the dura owing to the adhesion. The herniated disk increasingly encroached on the dura, slowly inducing reactive hypertrophy around the defect until it finally penetrated into the intradural space. Considering the chronic process in our case, we should be aware that an EDH may develop into an IDH.

Based on our experience in this case, we recommend dural incision using the surgical microscope as the operative procedure for IDH. Anterior lumbar interbody fusion (ALIF) was reported as another alternative in the treatment of IDH that can avoid unnecessary retraction of the nerve root and dura [6]. However, ALIF is limited when IDH is preoperatively diagnosed, and primary suture of the dural defect may be more difficult because vertebral bodies become an obstacle. In previous reports, there is general agreement that primary suture of the dural defect should be performed [1, 3, 5, 18]. Dural incision using the surgical microscope permitted a very clear visual field, allowing a careful dissection of disk fragments away from the adherent nerve roots and, finally, the repair of the ventral dural defect, while minimizing injuries to nerve roots.
